# Dicarbonyl stress in clinical obesity

**DOI:** 10.1007/s10719-016-9692-0

**Published:** 2016-06-24

**Authors:** Jinit Masania, Malgorzata Malczewska-Malec, Urszula Razny, Joanna Goralska, Anna Zdzienicka, Beata Kiec-Wilk, Anna Gruca, Julita Stancel-Mozwillo, Aldona Dembinska-Kiec, Naila Rabbani, Paul J. Thornalley

**Affiliations:** 1 Clinical Sciences Research Laboratories, Warwick Medical School, University of Warwick, University Hospital, Coventry, CV2 2DX UK; 2 Department of Clinical Biochemistry, Jagiellonian University Medical College, Krakow, Poland; 3 Warwick Systems Biology Centre, Senate House, University of Warwick, Coventry, CV4 7AL UK

**Keywords:** Methylglyoxal, Glycation, Glyoxalase, Obesity, Insulin resistance, Glyceroneogenesis, Hypoxia, Inflammation, type 2 diabetes, cardiovascular disease

## Abstract

The glyoxalase system in the cytoplasm of cells provides the primary defence against glycation by methylglyoxal catalysing its metabolism to D-lactate. Methylglyoxal is the precursor of the major quantitative advanced glycation endproducts in physiological systems - arginine-derived hydroimidazolones and deoxyguanosine-derived imidazopurinones. Glyoxalase 1 of the glyoxalase system was linked to anthropometric measurements of obesity in human subjects and to body weight in strains of mice. Recent conference reports described increased weight gain on high fat diet-fed mouse with lifelong deficiency of glyoxalase 1 deficiency, compared to wild-type controls, and decreased weight gain in glyoxalase 1-overexpressing transgenic mice, suggesting a functional role of glyoxalase 1 and dicarbonyl stress in obesity. Increased methylglyoxal, dicarbonyl stress, in white adipose tissue and liver may be a mediator of obesity and insulin resistance and thereby a risk factor for development of type 2 diabetes and non-alcoholic fatty liver disease. Increased methylglyoxal formation from glyceroneogenesis on adipose tissue and liver and decreased glyoxalase 1 activity in obesity likely drives dicarbonyl stress in white adipose tissue increasing the dicarbonyl proteome and related dysfunction. The clinical significance will likely emerge from on-going clinical evaluation of inducers of glyoxalase 1 expression in overweight and obese subjects. Increased transcapillary escape rate of albumin and increased total body interstitial fluid volume in obesity likely makes levels of glycation of plasma protein unreliable indicators of glycation status in obesity as there is a shift of albumin dwell time from plasma to interstitial fluid, which decreases overall glycation for a given glycemic exposure.

## The obesity epidemic and related complications

In the last 30 years the causes of global premature death and loss of productive life, as assessed in disability adjusted life years reported by the World Health Organization, has changed from communicable diseases in children towards non-communicable diseases in adults. Impaired metabolic health – development of insulin resistance leading to type 2 diabetes (T2DM) is now a leading cause of disability-adjusted life years. A major risk factor for this is being overweight and obese (body mass index >25 kg/m^2^) in which insulin resistance is a common feature and contributor to development of T2DM [1]. The number of people who are overweight and obese worldwide is now >2.1 billion, a prevalence of ca. 37 % in the adult population and 13 % in adolescents and children in developing countries and 23 % in developed countries [2]. In 2010, overweight and obesity were estimated to cause 3.4 million deaths worldwide. Most deaths attributable to overweight and obesity are cardiovascular deaths; 36 % of the increased risk of coronary heart disease (CHD) mortality and 59 % of the increased risk of stroke mortality risk associated with obesity was linked to increased blood pressure and cholesterol, 14 % and 25 % of CHD and stroke excess mortality were directly linked to glucose and the remaining excess risk is unexplained [3]. A further complication of overweight and obesity is non-alcoholic fatty liver disease (NAFLD) leading to hepatic steatosis (NASH), cirrhosis, liver failure and cancer [4]. Obesity-induced insulin resistance is a major driver of development of T2DM and NAFLD. NAFLD affects 20–40 % of the population in Westernised countries and will likely increase direct and indirect medical costs by 25 % in the next 5 years. There is an urgent requirement for improved understanding of the risk factors of insulin resistance, obesity and NAFLD and to guide interventions to decrease incidence and health impact.

## Obesity and the glyoxalase system

Recent reviews have described the impact of obesity on the glyoxalase system [5–8]. Herein consideration of the subject is advanced with description of recent experimental studies and findings with discussion of new underlying concepts, interpretation and inferences.

**Fig. 1:**
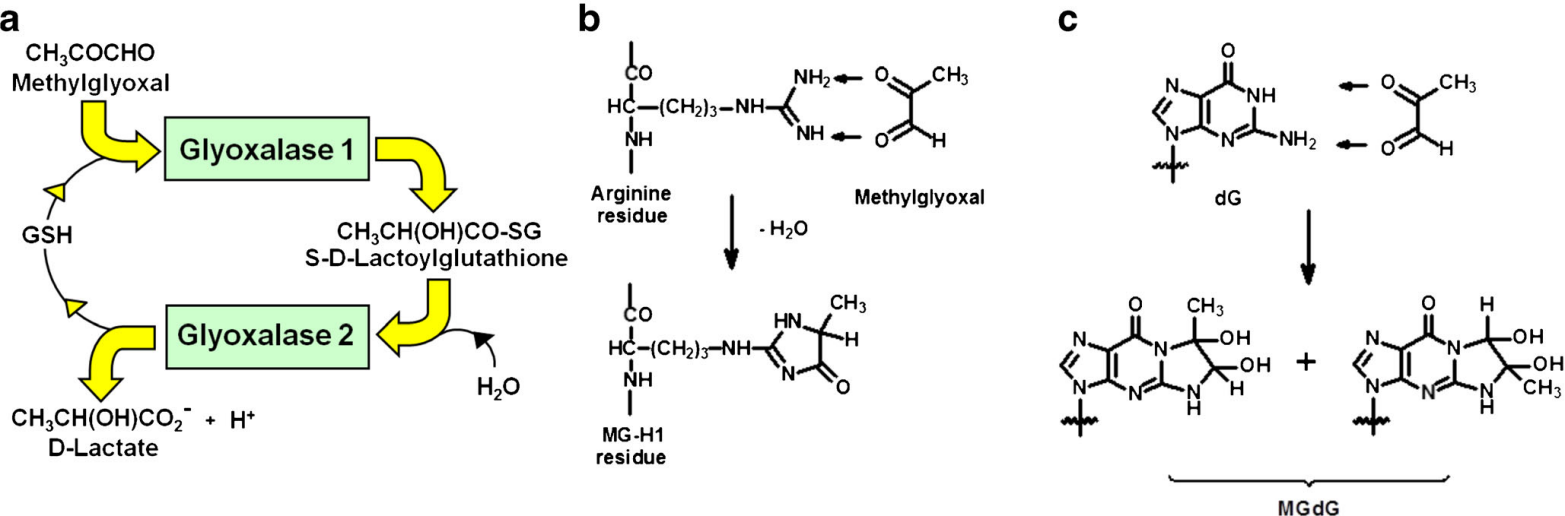
Biochemistry of dicarbonyl stress. **a** Metabolism of MG by the glyoxalase system. **b** Formation of hydroimidazolone MG-H1 from arginine residues. **c** Formation of imidazopurinone MGdG in DNA. Adduct residue is shown with guanyl base only

Genetic factors, dietary factors and early-life nutrition influence risk of insulin resistance and obesity. A gene functionally linked to obesity and diabetes is the glyoxalase 1 (Glo-1) gene, GLO-1 [9]. Glo-1 is part of the cytosolic glyoxalase system present in the cytoplasm of all mammalian cells – Fig. 1a. The glyoxalase system catalyses the metabolism of methylglyoxal (MG) to D-lactate via the intermediate S-D-lactoylglutathione and thereby suppresses the spontaneous modification of proteins and DNA by MG forming advanced glycation endproducts (AGEs). MG is the major precursor of AGEs *in vivo*, modifying mainly arginine residues in proteins to form hydroimidazolone MG-H1 - Fig. 1b, and deoxyguanosine residues in DNA to form imidazopurinones MGdG [10, 11] - Fig. 1c. Accumulation of MG adducts would otherwise cause protein dysfunction and mutagenesis. The function of the glyoxalase system is the enzymatic defence against MG glycation where Glo-1 catalyses the key step of removing the potentially damaging MG. Periods of increased MG concentration are called “dicarbonyl stress” produced by increased MG formation and/or decrease Glo-1 activity.

In human subjects, GLO-1 was linked to anthropometric measurements of obesity - upper-arm circumference and supra-iliac skinfold thickness [12]. In mice, meta-analysis of 34 mouse cross-breeding experiments linked GLO-1 to body weight [13]. Mice with a preference for a high energy-rich diet without marked health impairment have a relatively high expression of Glo-1 [5]. In the mouse overeating model of obesity, leptin mutant (ob/ob) mice, Glo-1 protein was decreased 80 % in the liver [14]. Recent conference reports described increased weight gain on high fat diet (HFD)-fed mouse with through-life expression of GLO-1 siRNA and mild Glo-1 deficiency, compared to wild-type controls [15], and decreased weight gain in Glo-1 overexpressing transgenic mice [16], suggesting a functional role of Glo-1 and dicarbonyl stress in obesity. We found increased MG concentration in hepatocyte-like hepatoma G2 cells *in vitro* incubated with saturated fatty acid and mono-unsaturated fatty acid, palmitic acid and oleic acid, respectively, suggesting that fatty acid metabolism may drive increased MG formation [17] – see below. HFD-fed wild-type mice had increased MG-H1 content of heart and liver, as judged by immunoassay [18]. Dicarbonyl stress may be a mediator of obesity and insulin resistance and thereby a risk factor for development of T2DM and NAFLD. Moreover, in a mouse model of hepatocellular carcinoma, Glo-1 was a tumour suppressor protein [19]. Hence, decrease of Glo-1 activity and hepatic dicarbonyl stress in NAFLD with progression to NASH may also increase risk of hepatocellular carcinoma.

## Obesity and dicarbonyl stress

Several studies have attempted to model dicarbonyl stress in obesity by administration of exogenous MG. Difficulties performing such studies are: (i) lack of commercial availability of suitable high purity MG, (ii) interference-free assay of MG, and (iii) and judgement of an appropriate dose to administer. Commercial 40 % MG contains 9–17 mol% formaldehyde and many other compounds that potentially interfere in studies of dicarbonyl stress [20]. Methods for preparation of high purity MG and interference-free assay of MG have been described [21, 22]. The flux of endogenous formation of MG has been estimated at ca. 3–6 mg/kg (ca. 0.05 % glycolytic rate, which we find relatively constant in many cell types) [23]. Experimental studies have often used 10–20 fold higher than this – which is likely similar to and exceeds the upper limit of severe dicarbonyl stress of poorly-controlled clinical diabetes and end stage renal disease [24, 25]. MG formation of cells with GLUT1 glucose transport increased only 2–3 fold in the high glucose concentration characteristic of T2DM and MG concentration in blood of patients with T2DM showed a similar 2–3 fold increase [24, 26].

Infusion of MG (60 mg/kg/day) into healthy rats induced impaired glucose tolerance, decreased glucose transporter GLUT-4, phosphoinositide-3-kinase activity, and insulin-stimulated glucose uptake in adipose tissue [27]. Administration of exogenous MG (50–75 mg/kg, daily, i.p.) induced insulin resistance in mice [28], inhibited insulin-stimulated phosphorylation of protein kinase B and extracellularly-regulated kinase, contributing to insulin resistance in muscle cells [29]. It also inhibited insulin-induced insulin receptor substrate tyrosine phosphorylation and phosphatidylinositol 3-kinase/protein kinase B pathway activation in pancreatic beta-cells [30], increased free fatty acids, hypoadiponectinemia, hypoxia and macrophage recruitment of adipose tissue [31]. These levels of MG exposure also arrested growth of rats, impaired renal function, induced hypercholesterolaemia and impaired vasodilation. There were also degenerative changes in cutaneous capillaries with loss of endothelial cells, basement membrane thickening, luminal occlusion and inflammatory response – increased receptor for AGE (RAGE), interleukin-1ß, tumour necrosis factor-α and connective tissue growth factor in medial layers of arteries, and transforming growth factor-ß in glomerular tufts, tubular epithelial cells and interstitial endothelial cells [32]. These MG administration models to date, therefore, explore features of MG intoxication. Some of the features produced may be similar to those developing in obesity – although they are likely markedly less severe.

## Moderate dicarbonyl stress in clinical obesity

To investigate dicarbonyl stress in clinical obesity we recruited obese and non-obese healthy human subjects and placed them on an isocaloric diet for 2 weeks. Blood samples were collected after overnight fasting and plasma prepared. Plasma MG was determined by stable isotopic dilution analysis liquid chromatography-tandem mass spectrometry (LC-MS/MS) [22] and plasma D-lactate concentration by endpoint enzymatic assay [33]. Plasma MG was increased 35 % in obese subjects and D-lactate was increased ca. 2-fold – Table 1. Plasma MG levels were intermediate between those found in non-obese healthy subjects and patients with type 1 or type 2 diabetes [22, 34, 35], suggesting clinical obesity is a state of moderate dicarbonyl stress. Plasma D-lactate levels, a surrogate and qualitative indicator of MG flux, also supported this [24, 35, 36] and suggest the flux of formation of MG is increased in obesity. (D-Lactate is metabolised in human subjects [37]). The MG-H1 residue content of plasma protein was determined in obese and non-obese subjects by exhaustive enzymatic hydrolysis of plasma protein and MG-H1 detection and quantitation by and stable isotopic dilution analysis LC-MS/MS [38] and no significant difference was found.

**Table 1:** Subject characteristics

Variable	Non-obese	Obese
N	18	29
Age (years)	50 ± 10	48 ± 11
Gender (M/F)	3/15	7/22
BMI (kg/m^2^)	27.8 ± 1.3	34.3 ± 3.3***
Systolic BP (mmHg)	125 ± 10	133 ± 21
Diastolic BP (mmHg)	79 ± 10	86 ± 10*
Hypertension (Y/N)	4/14	13/16
Fasting plasma glucose (mM)	5.1 ± 0.6	5.2 ± 0.6
Plasma MG (nM)	181 ± 61	245 ± 123*
Plasma protein MG-H1 (mmol/mol arg)	0.266 ± 0.105	0.264 ± 0.087
Plasma D-lactate (μM)	6.5 (2.4–13.7)	15.9 (10.0–20.2)*

Data are mean ± SD or median (lower – upper quartile). Significance: * and ***, *P* < 0.05 and *P* < 0.001, respectively (t-test for parametric data or Mann-Whitney U test for non-parametric data). Healthy human subjects were recruited at the Department of Clinical Biochemistry, Jagiellonian University Medical College, Krakow, Poland, after written informed consent. Inclusion criteria were: age 25–65 years, BMI - > 30–40 kg/m^2^ **(**obese) or <30 kg/m^2^ (all except one were overweight). Exclusion criteria were any co-morbidity and any medication for dysglycaemia or dyslipidaemia. Subjects were placed on a diet of 2300–2400 kcal/day (isocaloric diet) for 2 weeks prior to blood sampling. Peripheral venous blood samples were collected using EDTA as anticoagulant. Blood cells were sedimented by centrifugation (2000 g, 10 min) and plasma removed and retained for analysis. The study was approved by Jagiellonian University Bioethical Committee (Ref. KBET/82/B/2009 of 25 June 2009). Plasma and red blood cells were stored at -80 °C until analysis. The experiments conformed to the principles set out in the WMA Declaration of Helsinki

Estimates of MG and in white adipose tissue (WAT) in experimental models of overfeeding, HFD fed mice, were ca. 5000 nmol per g tissue *versus* ca. 2000 nmol per g tissue in controls – equivalent to ca. 5 mM and 2 mM MG, respectively [39]. Our estimates of MG in WAT of the same experimental model by the reference protocol [22] are markedly lower (mean ± SD): 2.91 ± 0.98 (*n* = 7) *versus* 1.47 ± 0.65 (*n* = 8, *P* < 0.01); equivalent to ca. 3 and 1.5 μM, respectively. We also found markedly lower levels of glyoxal in WAT but similar levels of 3-deoxyglucosone (Masania, J., Rabbani, N., Rossmeisl, M., Kopecky, J. and Thornalley, P.J.; unpublished observations). Mathematical modelling of MG formation and protein glycation in mouse tissue predicted MG concentrations in the 1–2 μM range in normal metabolism and are likely increased 2–3 fold in obesity [22]. Hence markedly higher estimates are not sustainable metabolically and may have been caused by interference – MG and glyoxal formation in pre-analytic processing. Trichloroacetic acid deproteinization and azide blocking of acid-stable peroxidase is required to avoid overestimation of MG and glyoxal in mouse tissues [40]. This requires further investigation.

## Plasma protein glycation adducts as reporters of glycation exposure in obesity

### Glycated albumin

**Fig. 2:**
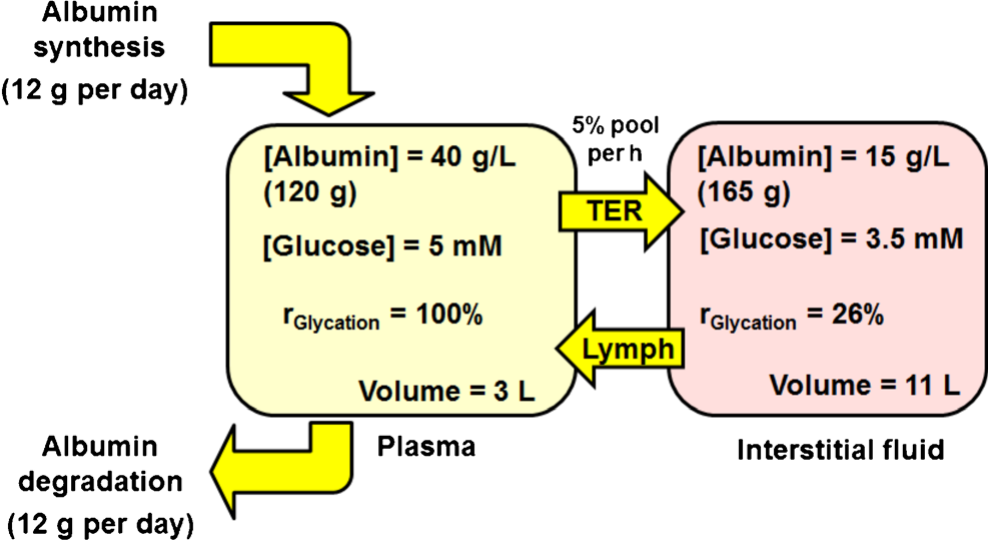
Glycation of albumin by glucose – glycation kinetics and dynamics in vascular and extravascular compartments. Physiological data from [45, 46]. Relative glycation kinetics deduced from: r_Glycation_ = k [Glucose][albumin], k is the glycation rate constant and assuming r_Glycation_ in the plasma compartment =100 %

In clinical obesity as insulin resistance develops, there is progressive decline in glycemic control with impaired fasting and postprandial hyperglycemia – as detected by continuous glucose monitoring. There is a moderate increase in glycated hemoglobin (A1C) as prediabetes develops [41] but surprisingly there is often a decrease in glycated albumin with increased BMI although A1C is increased. This is found both in obese adults and children [42, 43] and lower than expected glycated albumin in obesity also extends into the patients with T2DM [43]. The steady-state level of albumin glycation in plasma depends on the increased glucose concentration in plasma and duration over which it occurs, and also the residence time of albumin in the plasma compartment – as judged by the albumin transcapillary escape rate (TER). Until degraded with a half-life of ca. 20 days [44], albumin cycles from plasma into interstitial fluid, lymph and returns to plasma – with some leakage through renal glomeruli and return to venous circulation by the renal albumin retrieval pathway [45]. The rate of glycation by glucose normally is 4-fold higher in the plasma compared to interstitial fluid so that decrease in dwell time in plasma by increased TER may decrease glycation without change in plasma glucose concentration [45, 46] – Fig. 2. The rate of glycation of albumin by glucose, r_Glycation_, is directly proportional to the concentration of glucose and the concentration of albumin; r_Glycation_ = k_Glycation_ [Glucose][Albumin], where k_Glycation_ is the rate constant for glycation of albumin by glucose. The concentrations of albumin and glucose are 2.7-fold and 1.4 fold higher in plasma than interstitial fluid, which multiplied together indicate that the rate of glycation by glucose normally is 3.8 fold or ca. 4-fold higher in the plasma compared to interstitial fluid. Albumin TER is increased by hypertension (from 5.6 % per hour to 7.6 % per h) and plasma volume decreases by up to 10 % [47]. It is also increased in overweight/obese subjects with metabolic syndrome [48]. Obesity also increases the total body interstitial fluid volume [49]. This suggests that the explanation for decreased plasma glycated albumin in obesity with increased glucose exposure, as indicated by decreased glycated albumin/A1C ratio, may be due to a shift of albumin residence time from plasma to interstitial fluid in favour of the latter. This suggests glycated albumin is an unreliable marker of glycemic control in obesity.

The rate of degradation of albumin in obesity and diabetes may also influence the level of glycated albumin if changed. In obese subjects and patients with diabetes, with normal renal function, the synthesis of albumin and plasma concentration of albumin are unchanged from those of lean healthy controls, suggesting that the rate of degradation of albumin is also normal [50, 51]. In experimental diabetes early studies of Baynes and co-workers and others showed that the rate of degradation of albumin was not increased but slightly lower in streptozotocin-induced diabetic rats than in healthy controls [52, 53]. So there is no evidence that an increased rate of albumin degradation that contributes to decreased glycated albumin or AGE-modified albumin in obesity and diabetes. This may change, however, with impaired renal function with increased urinary loss of albumin and compensatory increased albumin synthesis [54].

## AGEs in plasma/serum protein in obesity

A shift of albumin residence time from plasma to interstitial fluid may also influence AGEs. Nε-Carboxymethyl-lysine (CML) residue content of serum protein was inversely linked to BMI and body fat mass [55, 56]. In studies where plasma early glycated (glycated albumin), CML and florescent AGE content were determined, all were decreased in obese subjects compared to controls [57]. Decrease of CML residue content of plasma protein in obesity was confirmed in an independent study and association with central obesity and inflammation [58]. Decreased CML residue content of plasma protein in obesity has been explained as due to enlargement of adipose tissue mass in obesity [59] and a contributory feature to this is likely decreased glycation of albumin due to the shift of albumin from plasma to interstitial fluid. An association with inflammation is also expected as albumin TER increases with increased capillary permeability in vascular inflammation [60]. Decreased residence time of albumin in the vascular lumen in obese subjects may also explain herein how plasma MG is increased without increase in MG-H1 residue content of plasma protein in our study – Table 1. This is supported by a recent study with a Glo-1 inducer where plasma MG concentration and whole body endogenous formation of MG-H1 adduct flux was decreased without decrease in plasma protein MG-H1 residue content. This was attributed to improved vascular function, decreased albumin TER and increased plasma dwell time of albumin with Glo-1 inducer intervention [61]. Given this, it is also likely that plasma protein AGE content in obesity is not a reliable indicator of AGE tissue exposure – as found previously [59].

## Source of dicarbonyl stress in obesity and its likely effects

**Fig. 3:**
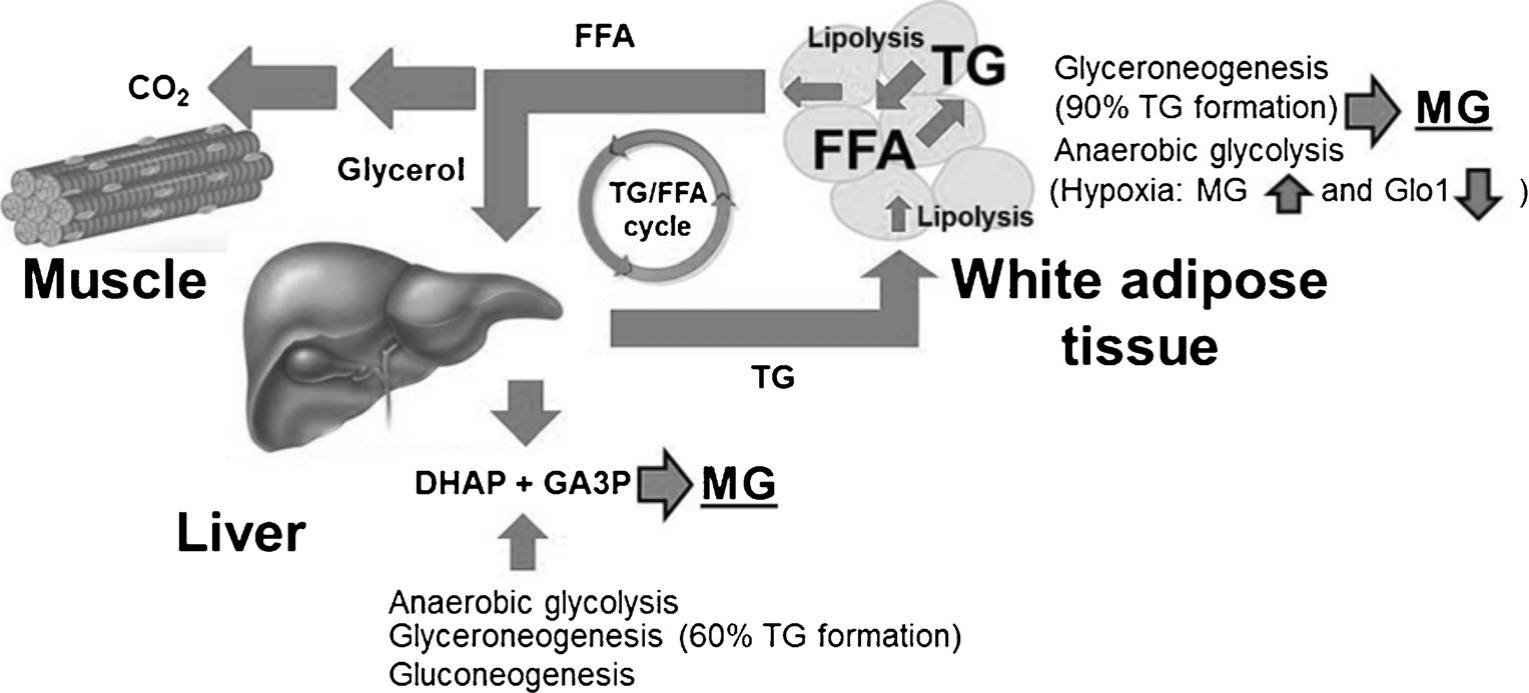
Increased formation of methylglyoxal in the triglyceride/free fatty acid cycle. Percentage flux of glyceroneogenesis in triglyceride formation in liver and adipose tissue is from [64]

Our finding of increased plasma MG and D-lactate concentrations in obese human subjects compared to non-obese subjects on an isocaloric diet suggests the source of dicarbonyl stress in obesity is not of dietary origin. Indeed, recent studies of metabolic transit of MG indicate that dietary MG is metabolised and/or reacts with protein in the intestinal lumen and has limited bioavailability [62]. Moreover the insulin resistance in obesity suggests the usually dominant source of MG formation from increased flux through anaerobic glycolysis may not be increased. Triosephosphates, however, are not limited to intermediates of anaerobic glycolysis but are also intermediates of glyceroneogenesis and gluconeogenesis. Increased glyceroneogenesis is associated with adipocyte expansion in obesity supporting increased fatty acid esterification for triglyceride deposition [63]. Most triglyceride synthesis involves glyceroneogenesis via triosephosphate intermediates [64]. Glyceroneogenesis is not limited, however, because it uses pyruvate as the carbon source, which has unrestricted entry and metabolism into major cell types experiencing insulin resistance – such as adipocytes, hepatocytes and skeletal muscle cells. As flux of glyceroneogenesis increases to support triglyceride synthesis, there is proportionate increase in MG formation by related increase in flux of triosephosphate formation. Most triosephosphate intermediates go on to form glycero-l-3-phosphate but with 0.05 % of triosephosphate degrading to MG, when the flux through glyceroneogenesis increases then concomitantly the flux of MG formation also increases. Increased glyceroneogenesis, therefore, is likely a major driver of increased MG formation in obesity – Fig. 3.

There is no inconsistency with previous findings of increased MG from red blood cells in high glucose concentration as therein MG is formed from increased concentrations of triosephosphates driven by increased uptake and metabolism of glucose in anaerobic glycolysis. Red blood cells do not suffer impaired glucose metabolism in insulin resistance. MG is mainly formed non-enzymatically from the same precursors in all cells and tissues – triosephosphates – but the pathways that sustain triosephosphate concentrations differ and may be multiple in cells and tissues.

In experimental models of obesity there is evidence of decreased Glo-1 activity in visceral adipose tissue [16]. Human Glo-1 protein is a dimer of 42 kDa. It undergoes post-translational modifications: C139 may form a mixed disulfide with GSH, inhibiting Glo-1 activity *in vitro***.** Glo-1 may be S-nitrosylated on C139 and is a substrate for calcium, calmodulin-dependent protein kinase II, with phosphorylation at T107. There is acetylation at K148 [65] and likely de-acetylation by cytosolic sirtuin-2 [66]. Glo-1 down regulation in obesity may be driven through hypoxia signalling by hypoxia-inducible factor-1α (HIF1α), which down-regulates Glo-1 expression [67]. As adipose tissue expands, interstitial oxygen tension decreases. HIF1α protein is highly enriched in expanding adipocytes as it drives increased adipose tissue vascularization. Adipocyte-specific deletion of HIF1α decreased HFD–induced adipose tissue inflammation and insulin resistance [68]. Increased MG formation by glyceroneogenesis and decreased Glo-1 expression through HIF1α signalling therefore provides the conditions for dicarbonyl stress in obesity.

The consequences of dicarbonyl stress in white adipose tissue (WAT) are unknown but contribute to insulin resistance. Obesity is associated with decreased activity of the insulin sensitising effects of fibroblast growth factor-21 (FGF21) due to down regulation of the FGF21 receptor cofactor β-Klotho [69]. Decreased FGF21 activity also impairs uncoupling protein-1 expression in brown adipose tissue, energy utilisation for thermogenesis [70] and facilitating fat deposition and weight gain. MG-driven protein glycation decreased expression of β-Klotho [71] and thereby likely contributes to insulin resistance. Decreased β-Klotho is also permissible for induction of pro-inflammatory mediators, interleukin-8, monocyte chemotactic protein-1, intracellular adhesion molecule-1 and receptor for AGEs, RAGE [71]. Overexpression of Glo-1 prevented insulin resistance and inflammation in HFD-fed mice, suggesting a functional role of dicarbonyl stress in obesity. This will be tested clinically by evaluation of Glo-1 inducer therapeutics.

In dicarbonyl stress there is increased protein glycation by MG. MG modification is directed to arginine residues often at functional sites of proteins and leads to functional change or inactivation. Protein targets of MG modification are called the dicarbonyl proteome [72]. The extent of protein glycation by MG is usually 1–2 % but low level increases can have profound physiological effect [73]. Examples are: MG modification of mitochondrial proteins, which increases formation of reactive oxygen species and oxidative damage [74]; modification of extracellular matrix proteins produces endothelial cell detachment with exposure of the sub-endothelium, platelet activation and thrombosis [72]; and modification of apolipoprotein B100 of low density lipoprotein (LDL) producing a atherogenic transformation to small, dense LDL [75]. Moreover, MG modification of proteins stimulates their proteolysis, decreasing protein half-life and thereby concentrations of the unmodified protein unless there is compensatory increased transcription - as found for apolipoprotein A1 of high density lipoprotein [76]. In relation to this, increasing endogenous MG by Glo-1 silencing in aortic endothelial cells changed expression of >400 genes [77]. Dicarbonyl stress, therefore, has proteome, dicarbonyl proteome and transcriptome signatures.

Increased MG formation from glyceroneogenesis on adipose tissue and liver and decreased Glo-1 activity in obesity likely drives dicarbonyl stress in WAT increasing the dicarbonyl proteome and related dysfunction. The functional significance of this is indicated by protection from insulin resistance, inflammation and weight gain in Glo-1 transgenic mice in experimental model of over-eating induced obesity. The clinical significance will likely emerge from on-going clinical evaluation of inducers of Glo-1 expression in overweight and obese subjects – for example, Clinicaltrials.gov; NCT02095873.

## Summary

MG metabolism and the glyoxalase system are disturbed in obesity leading to dicarbonyl stress. Functional genomics studies with Glo-1 in the overfeeding model of HFD-fed mice suggest dicarbonyl stress is a risk factor for health impairment and complications of obesity. Likely drivers of dicarbonyl stress in obesity are: increased formation of MG from increased glycerogenesis in triglyceride synthesis and decreased Glo-1 expression and activity through hypoxia and inflammatory signalling. A recent clinical intervention study with a Glo-1 inducer produced a profound improvement of insulin resistance, improved glycemic control and arterial function and decreased vascular inflammation, suggesting that Glo-1 inducer therapeutics may have a future key role in alleviating complications of obesity. Increased albumin TER in obesity associated with increased fat mass, hypertension and inflammation suggest glycated albumin is not a reliable measure of glycemic control and formation of AGEs in obesity.

## Abbreviations

A1C: Glycated hemoglobin
AGEs: Advanced glycation endproducts
CHD: Coronary heart disease
CML: Nε-Carboxymethyl-lysine
DHAP: Dihydroxyacetonephosphate
FFA: Free fatty acid
FGF21: Fibroblast growth factor-21
GA3P: Glyceraldehyde-3-phosphate
Glo-1: Glyoxalase 1
HFD: High fat diet
HIF1α: Hypoxia-inducible factor-1α
LC-MS/MS: Liquid chromatography-tandem mass spectrometry
MG: Methylglyoxal
MGdG: 3-(2′-deoxyribosyl)-6,7-dihydro-6,7-dihydroxy-6/7-methylimidazo-[2,3-b]purine-9(8)one
MG-H1: Nδ-(5-hydro-5-methyl-4-imidazolon-2-yl)-ornithine
NAFLD: Non-alcoholic fatty liver disease
NASH: Hepatic steatosis
RAGE: Receptor for advanced glycation endproducts
T2DM: Type 2 diabetes
TG: Triglyceride
TER: Transcapillary escape rate
WAT: White adipose tissue
